# Potentially inappropriate prescribing for people with dementia in ambulatory care: a cross-sectional observational study

**DOI:** 10.1186/s12877-024-04949-8

**Published:** 2024-04-10

**Authors:** Nahla A. Alageel, Carmel M. Hughes, Monira Alwhaibi, Walid Alkeridy, Heather E. Barry

**Affiliations:** 1 Primary Care Research Group, School of Pharmacy, Medical Biology Centre, Queen’s University Belfast, 97 Lisburn Road, BT9 7BL Belfast, UK; 2 Department of Clinical Pharmacy, College of Pharmacy, King Saud University, Riyadh, Saudi Arabia; 3 Department of Medicine, College of Medicine, King Saud University, Riyadh, Saudi Arabia; 4 Department of Medicine, Geriatric Division, University of British Columbia, Vancouver, Canada; 5 General Administration of Home Health Care, Therapeutic Affairs Deputyship, Riyadh, Saudi Arabia

**Keywords:** Ambulatory care, Dementia, Electronic health records, Inappropriate prescribing, Pharmacoepidemiology, Polypharmacy, Potentially inappropriate medication

## Abstract

**Background:**

Studies have shown that potentially inappropriate prescribing (PIP) is highly prevalent among people with dementia (PwD) and linked to negative outcomes, such as hospitalisation and mortality. However, there are limited data on prescribing appropriateness for PwD in Saudi Arabia. Therefore, we aimed to estimate the prevalence of PIP and investigate associations between PIP and other patient characteristics among PwD in an ambulatory care setting.

**Methods:**

A cross-sectional, retrospective analysis was conducted at a tertiary hospital in Saudi Arabia. Patients who were ≥ 65 years old, had dementia, and visited ambulatory care clinics between 01/01/2019 and 31/12/2021 were included. Prescribing appropriateness was evaluated by applying the Screening Tool of Older Persons Potentially Inappropriate Prescriptions (STOPP) criteria. Descriptive analyses were used to describe the study population. Prevalence of PIP and the prevalence per each STOPP criterion were calculated as a percentage of all eligible patients. Logistic regression analysis was used to investigate associations between PIP, polypharmacy, age and sex; odds ratios (ORs) and 95% confidence intervals (CIs) were calculated. Analyses were conducted using SPSS v27.

**Results:**

A total of 287 PwD were identified; 56.0% (*n* = 161) were female. The mean number of medications prescribed was 9.0 [standard deviation (SD) ± 4.2]. The prevalence of PIP was 61.0% (*n* = 175). Common instances of PIP were drugs prescribed beyond the recommended duration (*n* = 90, 31.4%), drugs prescribed without an evidence-based clinical indication (*n* = 78, 27.2%), proton pump inhibitors (PPIs) for > 8 weeks (*n* = 75, 26.0%), and acetylcholinesterase inhibitors with concurrent drugs that reduce heart rate (*n* = 60, 21.0%). Polypharmacy was observed in 82.6% (*n* = 237) of patients and was strongly associated with PIP (adjusted OR 24.1, 95% CI 9.0–64.5).

**Conclusions:**

Findings have revealed a high prevalence of PIP among PwD in Saudi Arabia that is strongly associated with polypharmacy. Future research should aim to explore key stakeholders’ experiences and perspectives of medicines management to optimise medication use for this vulnerable patient population.

**Supplementary Information:**

The online version contains supplementary material available at 10.1186/s12877-024-04949-8.

## Background

Dementia is a progressive syndrome caused by underlying neurodegenerative processes and characterised by a decline from a previously attained cognitive level that affects memory, thinking, behaviour and activities of daily living [1]. The demographic shifting toward ageing populations is generating significant increases in dementia prevalence. In 2019, the number of people living with dementia worldwide was 55 million and this number is projected to increase to 139 million by 2050 [1]. In Saudi Arabia, there are no accurate national data about the prevalence of dementia, although Middle Eastern countries are predicted to have one of the highest dementia prevalence estimations in the world by 2050 [2]. Dementia is a leading cause of death globally, and it has been estimated that Alzheimer’s disease and other dementias are one of the leading causes of death for females in Saudi Arabia [3]. Increasing age is the strongest known risk factor for dementia, with the incidence doubling with every five-year increment in age [1]. Ageing is also combined with a gradual decline in physical function and a growing risk of multiple chronic conditions that usually require prescribing of multiple medications (polypharmacy) [4, 5].

Polypharmacy has been described using a numerical threshold, commonly four or five medications [6, 7]. Given the high prevalence of comorbid medical conditions and frailty among people with dementia (PwD), the risks of polypharmacy, drug-drug and drug-disease interactions are greater than among their older counterparts [8–12]. Polypharmacy may increase the possibility of adverse drug reactions, reduced medication adherence, and potentially inappropriate prescribing (PIP) [13]. PIP is the prescribing of medications where the risk of potential harm exceeds the potential benefit, and a safer option is available to treat the condition [14]. It has been linked to negative consequences in older adults such as adverse drug events, hospitalisation, mortality, and increased healthcare costs [15–19]. Several validated assessment tools are available for measuring the appropriateness of prescribing in elderly patients. One of the most commonly used criterion-based tools is the Screening Tool of Older People’s potentially inappropriate Prescriptions (STOPP) criteria. STOPP consists of 81 criteria classified according to physiological body system to identify potentially inappropriate medications [20].

Additional challenges for PwD are the deterioration in cognitive function and communication abilities which may have a negative influence on medication adherence [21, 22]. Furthermore, up to 90% of PwD experience one or more non-cognitive symptoms which require prescribing of one or more psychoactive medications [23, 24]. For example, the prescribing of three or more psychoactive medications concurrently for more than one month was reported in 13.9% community dwelling PwD in the United States in 2018 [25]. In addition, medications such as anticholinergics and sedative hypnotics, have been associated with an increased risk of hospitalisation and death in PwD [26, 27].

Several epidemiological studies have assessed the appropriateness of prescribing for community dwelling PwD and have reported the prevalence of PIP between 56 and 73%. The most common instances of PIP involved antipsychotics, benzodiazepines, cardiovascular drugs, proton pump inhibitors (PPIs) and medications with anticholinergic activity [28–32]. While studies have explored the appropriateness of prescribing for older adults in Saudi Arabia, research focused on PIP for PwD has not been conducted [33–36]. Therefore, the aim of this study was to explore prescribing patterns and the appropriateness of medications prescribed for PwD in ambulatory care in a tertiary university hospital in Riyadh: King Saud University Medical City (KSUMC). Specific study objectives were to assess the number and types of medications prescribed to PwD in Saudi Arabia, estimate the prevalence of polypharmacy and PIP (through application of a subset of the STOPP criteria version 2), and investigate the associations between PIP and other patient-related factors such as age, sex, and polypharmacy.

## Methods

This study is reported according to the Strengthening the Reporting of Observational Studies in Epidemiology (STROBE) checklist (Additional File 1).

### Setting and data source

This study took place at KSUMC, which is a tertiary care teaching medical city affiliated with King Saud University (KSU) in Riyadh. It includes an outpatient department with more than 20 clinics including neurology, internal medicine, psychiatry, and other specialities. All services and care are provided free of charge for KSU staff, students, employees, and their families and other citizens who live in Riyadh or who are referred from other hospitals across Saudi Arabia.

KSUMC utilises electronic health records (EHRs) to store patient medical information (e.g. diagnostic test results) and a computerised physician order entry system to record patient-specific interventions during the medical encounter (e.g. prescribing of medication) by the clinician who has access to the patient’s EHR through the patient’s unique medical record number. The clinical information documented in EHRs can be recorded in a structured (e.g. blood analysis results) or unstructured (e.g. progress note) format by healthcare providers.

### Study design and population

This was a cross-sectional, retrospective study which included all older people (aged ≥ 65 years) diagnosed with dementia of any type during the study period (1st January 2019 to 31st December 2021) and followed up in an outpatient ambulatory clinic at KSUMC. The study was approved by the Institutional Review Board of KSU (reference number E-21-6288). The requirement for individual informed consent was formally waived by the Institutional Review Board of King Saud University because of the retrospective nature of this study and the data were analysed after anonymisation. All methods were carried out in accordance with relevant guidelines and regulations.

Study participants were identified by an electronic search of the EHR database, conducted by an informatics technician and the researcher (NA) using two approaches. Firstly, a search was conducted using codes for dementia and its related terms using the International Classifications of Diseases-10th edition, Clinical Modification (ICD-10-CM), which is the disease coding system used by KSUMC. The researcher assessed EHRs identified in this way and a geriatric physician (WA) was consulted if there was any ambiguity. As we anticipated that diagnostic coding may not always be accurate, we utilised a second approach to identify participants based on prescribing of a dementia medication. This identified all individuals who were dispensed any medication used for the management of dementia (i.e. donepezil, galantamine, rivastigmine, memantine) from the outpatient pharmacy records during the study period. The resultant list of participants’ medical record numbers was checked for duplication by the researcher and a unique list of study participants was obtained. Patients who were aged less than 65 years or who died during the study period were excluded. All data were anonymised by the researcher, and the research team had no access to any patient identifiable data. All data were extracted from EHRs by the researcher using a structured data collection form and included patients’ age, sex, clinical conditions (to assist with application of specific STOPP criteria), biochemical data and clinical parameters (sodium, potassium, calcium, and creatinine levels, estimated Glomerular Filtration Rate, blood pressure, arterial blood gases), and prescribed medications.

### Measurement of potentially inappropriate prescribing using the STOPP criteria

PIP was assessed using STOPP Criteria. Out of 81 STOPP criteria, all except three were applied due to either the medication being unavailable at the hospital (‘*ticlopidine in any circumstances*’) or due to difficulty in assessing that criterion from the EHR because it required frequent patient follow up while patients in a tertiary hospital setting are usually followed every three to six months (‘*beta-blockers in diabetes mellitus with frequent hypoglycemic episodes*’, and ‘*vasodilator drugs with persistent postural hypotension, i.e. recurrent drop in systolic blood pressure ≥ 20mmHg*’). STOPP criteria which stated to avoid chronic use, e.g. ‘*long term use of non-steroidal anti-inflammatory drugs (NSAIDs) > 3 months*’, were evaluated by identifying patients who used the drug(s) for durations exceeding three months within the study period. STOPP criteria which specified a drug or drug class not to be used with a specific electrolyte imbalance were assessed by checking the patient’s laboratory values during the time the medication was prescribed, e.g. ‘*thiazide diuretic with current significant hypokalaemia, hyponatremia, or hypercalcemia*’. STOPP criteria that recommended a specific daily dose not to be exceeded for a medication, such as ‘*oral elemental iron doses greater than 200 mg daily*’, were assessed by manually calculating the total daily dosage for each patient prescribed this medication.

### Exposures

All study participants were classified by sex, age group, and type of dementia. Participants’ medication use was evaluated by the researcher; all medications prescribed for patients during the study period were recorded and the total number of chronic medications (i.e. those prescribed for ≥ 3 months) was counted for each patient.

### Polypharmacy

In this study, polypharmacy was identified if a patient was prescribed five or more chronic medications concurrently during the study period. Chronic medications were classified according to the Anatomical Therapeutic Chemical (ATC) classification system of the World Health Organization (WHO) [37].

### Outcomes

The primary outcome was the overall prevalence of PIP according to the STOPP criteria in PwD in ambulatory care at KSUMC. Secondary outcome measures were the prevalence of polypharmacy; the types of medications prescribed to PwD; the prevalence of PIP per each STOPP criterion; and the association between PIP, age group, sex, and polypharmacy.

### Statistical analysis

Descriptive statistics were used to describe the study population. Percentage estimates were calculated for both the total prevalence of PIP in the study population and the prevalence per each STOPP criterion. Bivariate analyses were used to confirm the significance of association of polypharmacy (categorised as 0–4 versus ≥ 5 chronic medications, age (65–74, 75–84, 85–94, ≥ 95 years) and gender (male, female) with PIP (any versus none), and these were included in the multivariable regression model; adjusted odds ratios (OR) and 95% confidence intervals (CI) were calculated. Statistical significance was set at *P*-value equal to or less than 0.05. Analyses were performed by the researcher using IBM SPSS for Windows Software Package, Version 27 [38].

## Results

### Identification of study participants

Three hundred and thirty-three patients were identified through ICD-10 dementia codes and 270 patients were identified through outpatient pharmacy records (*n* = 603 patients in total). Following removal of duplicates (*n* = 87), 516 patients were assessed against the study eligibility criteria. Two hundred and twenty-nine patients were excluded: four patients were aged < 65 years, 139 died during the study period; 45 did not have a confirmed diagnosis of dementia, and 41 were not followed up in the outpatient setting. Therefore, the total number of eligible PwD identified during the study period was 287.

### Characteristics of the study population

The mean age of study participants was 78.8 [standard deviation (SD) ± 8.0] years and the majority were female (*n* = 161, 56.0%; Table 1).

**Table 1:** Characteristics of the study population (*N* = 287)

Variables	N (%) 287 (100.0)
Sex	
Female	161 (56.0)
Age (years)	
65–74	87 (30.3)
75–84	130 (45.3
85–94	61 (21.3)
≥ 95	9 (3.1)
Dementia types	
Alzheimer’s disease	210 (73.2)
Vascular dementia	41 (14.3)
Mixed dementia	21 (7.3)
Dementia with Lewy bodies	11 (3.8)
Frontotemporal dementia	4 (1.4)
Polypharmacy (≥ 5 chronic medications)	
Yes	237 (82.6)
PIP	
Yes	175 (61.0)
Number of PIMs prescribed	
0	112 (39.0)
1	71 (24.7)
2	40 (14.0)
3	37 (12.9)
≥4	27 (9.4)

PIM: potentially inappropriate medication; PIP: potentially inappropriate prescribing; SD: standard deviation

**Fig. 1:**
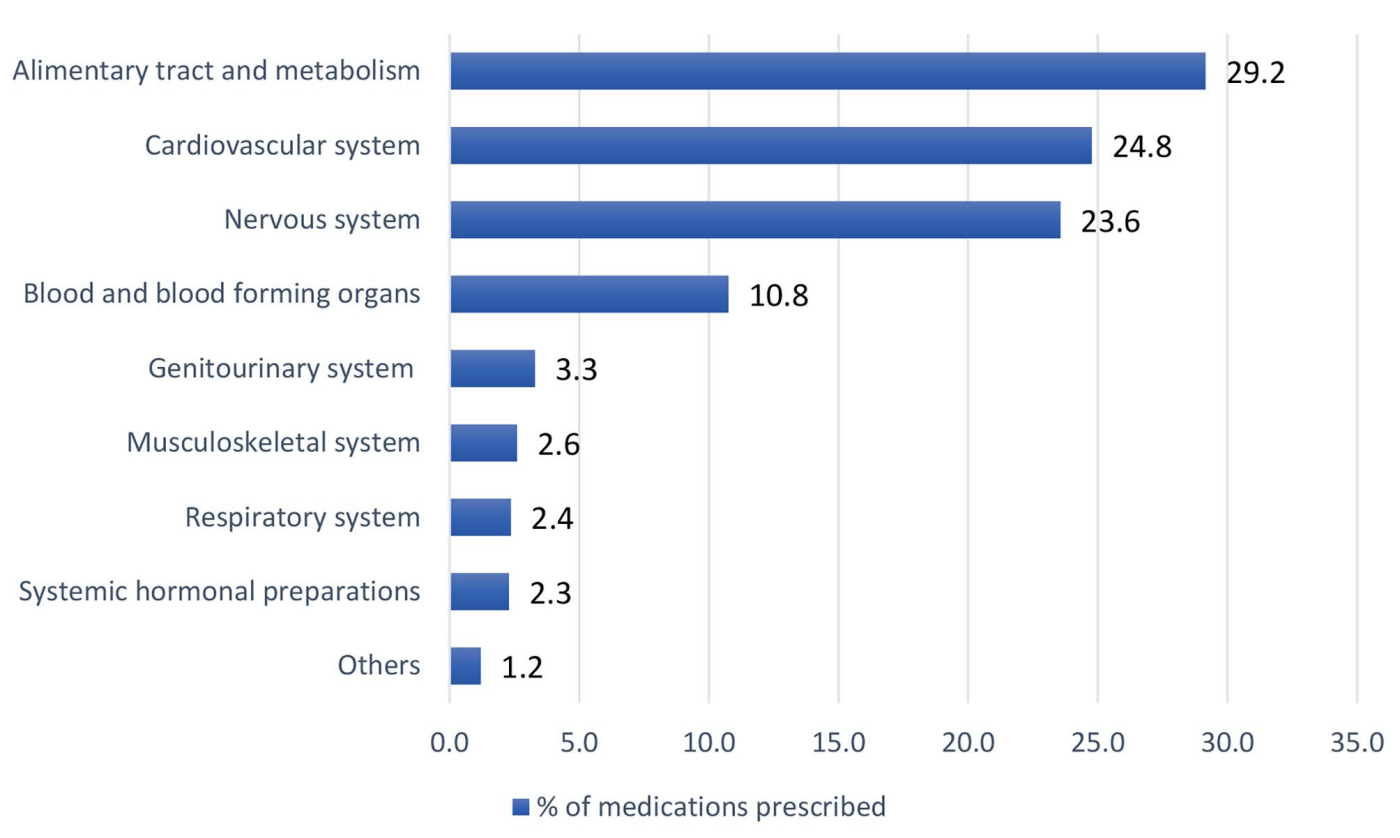
Percentage of medications prescribed to people with dementia during the study period according to Anatomical Therapeutic Chemical (ATC) classification

Alzheimer’s disease was the most common cause of dementia (*n* = 210, 73.2%) followed by vascular (*n* = 41, 14.3%) and mixed dementia (*n* = 21, 7.3%) respectively. Patients were taking a mean number of 8.9 (SD ± 4.2) chronic medications. Over three-quarters of patients (*n* = 237, 82.6%) were prescribed five or more chronic medications, whilst the use of ten or more chronic medications (excessive polypharmacy) was observed in almost half of patients (*n* = 139, 48.4%). A total of 2,576 chronic medications were recorded during the study period. According to the ATC classification, the most prescribed medications were the alimentary tract and metabolism class (29.0%), which comprise a high percentage of antidiabetic medications and PPIs, followed by cardiovascular (24.8%) and nervous system medications (23.6%; Fig. 1).

### Overall prevalence of PIP

The overall prevalence of PIP according to the STOPP criteria that were applied was 61.0% (*n* = 175). Almost one-quarter of the study population (*n* = 71, 24.7%) was prescribed one potentially inappropriate medication, 40 patients (14.0%) were prescribed two potentially inappropriate medications, 37 patients (12.9%) were prescribed three potentially inappropriate medications, and 27 patients (9.4%) were prescribed four or more potentially inappropriate medications.

### Prevalence of PIP according to individual STOPP criteria

The most common instance of PIP was *‘any drug prescribed beyond the recommended duration, where treatment duration is well defined’* (*n* = 90, 31.4%). The second most frequent instance of PIP was *‘drugs prescribed without an evidence-based clinical indication’* (*n* = 78, 27.2%). Table 2 describes the five most common instances of PIP. The prevalence for each STOPP criterion applied is presented in Additional file 2. *‘Proton pump inhibitors (PPI) for uncomplicated peptic ulcer disease or erosive peptic oesophagitis at full therapeutic dosage for > 8 weeks’* was prevalent in around one quarter of patients (*n* = 75, 26.0%), followed by acetylcholinesterase inhibitors with concurrent treatment with drugs that reduce heart rate (*n* = 60, 21.0%), anticholinergics/antimuscarinics in patients with delirium or dementia (*n* = 28, 9.8%), and antimuscarinic drugs with dementia, or chronic cognitive impairment or narrow-angle glaucoma or chronic prostatism (*n* = 25, 8.7%). Duplication of therapy within drug classes was found in 8.0% of the study population (*n* = 23), which was most frequently observed with calcium channel blockers (*n* = 6, 2.1%) and acetylcholinesterase inhibitors (*n* = 4, 1.4%), while dual antiplatelet therapy with aspirin plus clopidogrel as secondary stroke prevention was prevalent in 5.2% (*n* = 15). Many other STOPP criteria had a prevalence of less than 5.0%, such as *‘sulphonylureas with a long duration of action with type 2 diabetes mellitus’* and ‘*neuroleptic antipsychotics in patients with behavioural and psychological symptoms of dementia (BPSD)*’.

**Table 2:** Prevalence of most common instances of potentially inappropriate prescribing among 287 people with dementia

STOPP criterion description (potential risk)	Number of patients	% of patients
Any drug prescribed beyond the recommended duration, where treatment duration is well defined.	90	31.4
Any drug prescribed without an evidence-based clinical indication.	78	27.2
PPI for uncomplicated PUD or erosive peptic oesophagitis at full therapeutic dosage for > 8 weeks *(dose reduction or earlier discontinuation indicated)*	75	26.0
AchEIs with a known history of persistent bradycardia (< 60 beats/min), heart block or recurrent unexplained syncope or concurrent treatment with drugs that reduce heart rate such as beta blockers, digoxin, diltiazem, verapamil *(risk of cardiac conduction failure, syncope, and injury)*	60	21.0
Anticholinergics/ antimuscarinics in patients with delirium or dementia *(risk of exacerbation of cognitive impairment)*	28	9.8

AchEIs: acetylcholinesterase inhibitors; BP: blood pressure; PPI: proton pump inhibitor; PUD: peptic ulcer disease; STOPP: Screening Tool of Older Persons Potentially Inappropriate Prescriptions

### Factors associated with PIP following bivariate analysis

Following bivariate analysis, the presence of PIP was not found to be significantly different between males and females, or between different age groups (Table 3). However, PwD who received polypharmacy were significantly more likely to receive PIP compared to PwD prescribed less than five medications (*P* < 0.001).

**Table 3:** Bivariate analysis for the relationship between PIP and covariates*

	PIP		NO PIP		*P* value
	*N*	%	*N*	%	
Polypharmacy					
No	5	10	45	90	< 0.001
Yes	168	70.9	69	29.1	
Sex					
Male	76	60.3	50	39.7	0.544
Female	97	60.2	64	39.8	
Age group (years)					
65–74	54	61.4	34	38.6	0.21
75–84	84	65.1	45	34.9	
85–94	31	50.8	30	49.2	
≥ 95	4	44.4	5	55.6	

*% presented in this table are row percentage

### Factors associated with PIP in regression analysis

Multivariable logistic regression confirmed that polypharmacy was significantly associated with PIP (Table 4). Those receiving five or more chronic medications were more likely to be exposed to PIP compared to those on zero to four chronic medications (adjusted OR 24.1, 95% CI 9.0–64.5) after adjusting for sex and age. However, no significant associations were observed between PIP and age (after adjustments for sex and polypharmacy) or sex (after adjustments for age and polypharmacy).

**Table 4:** Logistic regression analysis investigating any PIP criteria

PIP	Unadjusted OR (95% CI)	Adjusted OR (95% CI)
Polypharmacy		
No (ref)	1	1
Yes	21.9 (8.3–57.5)	24.1 (9.0-64.5)
Sex		
Male (ref)	1	1
Female	0.9 (0.6–1.6)	0.8 (0.5–1.5)
Age group (years)		
65–74 (ref)	1	1
75–84	1.2 (0.7-2.0)	1.7 (0.9–3.3)
85–94	0.6 (0.3–1.2)	0.8 (0.4–1.7)
≥ 95	0.6 (0.2–2.4)	0.7 (0.2–3.3)

CI: confidence interval; OR: odds ratio; PIP: potentially inappropriate prescribing; ref: reference

## Discussion

In this study, we investigated the overall prevalence of PIP in PwD aged 65 years and over who visited an ambulatory care clinic during the period 2019–2021 in Saudi Arabia. Approximately two-thirds of the population received PIP, according to a subset of the STOPP criteria applied. Among the factors investigated, polypharmacy was significantly associated with PIP, while no association was observed with age or sex.

To the best of the authors’ knowledge, this is the first study to use the STOPP criteria to examine the appropriateness of prescribing for PwD in an ambulatory care setting in Saudi Arabia. The high prevalence of PIP reported in this population is somewhat consistent with findings reported by other international studies that have investigated the prevalence of PIP among community dwelling PwD using the STOPP criteria [27, 29, 30, 39, 40]. Moreover, our results are also comparable to findings from national studies which investigated the use of potentially inappropriate medications among older people who visited ambulatory care settings in Saudi Arabia, and which reported the prevalence of PIP between 52.5% and 61% [32, 34, 35]. However, direct comparison with these findings is difficult as these studies did not focus specifically on PwD and reported the prevalence of PIP using the Beers criteria.

The prevalence of polypharmacy amongst PwD in this study is slightly higher than the upper range of prevalence described for older people in the ambulatory care setting in Saudi Arabia (66.3–80.5%) and is comparable to other studies which have reported polypharmacy amongst PwD [12, 27, 34, 41]. Additionally, there was a strong association between polypharmacy and PIP which supports findings from earlier published studies [27, 34, 42–44].

The most commonly prescribed potentially inappropriate medication identified in this study was PPIs. The STOPP criteria recommend that when PPIs are prescribed for the treatment of uncomplicated peptic ulcer disease (PUD) or erosive peptic esophagitis at full therapeutic dosage, this should not exceed eight weeks in duration. Other indications for PPIs include patients on aspirin or non-steroidal anti-inflammatory drugs (NSAIDs) with a history of PUD or prescribed other medications that increase the risk of PUD simultaneously. However, we found most of the patients in this study who were prescribed these medications did not have a clear indication, and prescribing took place for longer than the recommended duration. This finding has been reported by other national and international epidemiological studies which have investigated PIP in older people or community dwelling PwD specifically [27, 29, 34, 35, 39, 45]. The long-term use of PPIs by older adults has been linked to an increased risk of osteoporosis, fractures, *Clostridium difficile* infection, and pneumonia [46, 47]. Nevertheless, when prescribed appropriately for the established indications, PPIs have a positive effect on the outcomes of patients with gastric-acid related disease.

One fifth of the study population was prescribed acetylcholinesterase inhibitors with concurrent treatment with drugs that reduce heart rate such as beta blockers or calcium channel blockers. This drug-drug interaction could lead to bradycardia, hypotension, or other cardiac conduction abnormalities that adversely affect the patient. Therefore, caution should be taken when these medications prescribed concomitantly, and patient-specific clinical judgment and monitoring are required to avoid the potential cardiac risk. Whilst the use of anticholinergics/antimuscarinic medications was observed at a lower prevalence (9.8%) compared with other studies, their use is not recommended in older people, and especially PwD, who may be sensitive to the cognitive side effects of these medications such as confusion, drowsiness, and hallucinations [27, 48]. Medications with anticholinergic activity are prescribed for a variety of indications including overactive bladder, chronic obstructive pulmonary disease, Parkinson’s disease, and allergies. The concomitant use of several medications with anticholinergic activity increases the burden of side effects (termed anticholinergic burden; ACB). A variety of scales have been developed to quantify ACB such as the Anticholinergic Cognitive Burden Scale, and the Anticholinergic Risk Scale [49]. High ACB is linked with negative outcomes such as decreased physical functioning, falls, and hospitalisation in PwD, thus interventions to limit anticholinergic drug prescribing and use by PwD are required [50, 51].

The potentially inappropriate use of antipsychotic medications amongst this patient sample was low. Whilst this is a reassuring finding given the risks associated with the use of these medications in people with dementia, it does not mean that prescribing of these medications was low overall. In contrast, it indicates appropriate prescribing of antipsychotics in a large proportion of PwD who either suffered from severe symptoms and/or for whom non-pharmacological approaches had failed, particularly for those at an advanced stage of the disease. Other studies conducted in Saudi Arabia have reported greater prevalence of prescribing of antipsychotic medications, although these studies focused on the older population generally and were not specific to people with dementia [33, 52]. Similar to the study by Meraya et al. [52], the use of benzodiazepines in our study population was low indicating judicious use of these medications in Saudi Arabia.

Several interventions have been designed and evaluated to reduce PIP among older adults in the ambulatory care setting. Medication reviews conducted by pharmacists, the use of computerised decision support systems, multifaceted interventions including educational outreach, and audit and feedback were found to be beneficial in improving prescribing appropriateness [53–55]. Conversely, deprescribing (‘the planned and supervised process of dose reduction or stopping unnecessary or potentially harmful medication’) may have beneficial effects on reducing prescribing of potentially inappropriate medications and the burden of polypharmacy [56, 57]. A growing body of evidence has shown that successful deprescribing interventions were multidisciplinary in nature, with many including the provision of patient educational materials [58]. Currently, most of the medication optimisation and deprescribing interventions for PwD which are described in the literature are of poor quality, have multiple methodological limitations, have only targeted certain medication classes, have focused on medication-related outcomes instead of patient-centred outcomes, or been restricted to inpatients or those residing in long-term care facilities [59–61]. Future research should focus on the development and evaluation of complex, multidisciplinary and theory-based interventions for PwD that can be implemented in ambulatory care settings and cover multiple medications instead of specific medication classes [59, 61].

It is anticipated that the findings from this study will add to the limited evidence base that currently exists on appropriateness of prescribing for PwD in Saudi Arabia. In highlighting areas where prescribing may be considered potentially inappropriate, this should draw healthcare providers’ attention to this issue, particularly during clinical encounters with this patient population and when planning medication reconciliation and review activities. Healthcare providers’ attitudes and willingness towards improving prescribing and use of medications for PwD in Saudi Arabia should be explored in future research, along with the views of PwD and their caregivers in Saudi Arabia, as key partners in medication management.

### Strengths and limitations

The present study has explored, for the first time, the prescribing patterns and appropriateness of prescribing for PwD in Saudi Arabia. The availability of medical diagnostic information and other clinical data through EHRs enabled us to apply a comprehensive set of STOPP criteria. However, it is important to consider the study’s limitations. Firstly, the STOPP criteria were assessed based on data extracted from EHRs, while medications prescribed by healthcare providers outside the hospital setting could not be captured. In addition, this study did not identify reports from participants’ family members or carers about patients’ self-medication, consider medications obtained without prescription (such as those bought over the counter), or medications prescribed “as needed”, which might have resulted in an underestimation of the prevalence of polypharmacy and PIP, especially for analgesic medications. Whilst the prevalence of prescribing of chronic NSAIDs was low in this study, a survey-based cross-sectional study using the Saudi National Survey for Elderly Health, which included around 3,000 Saudi older adults, reported that NSAIDs were used by 50% of the participants [62]. However, comparison is limited because PwD were not included in this survey. Moreover, other factors such as presence of comorbidities or recent hospitalisation, which may have had an impact on the prevalence of PIP, were not investigated in this study. We acknowledge some limitations to the approaches used to identify study participants– diagnostic codes may have been inaccurate and some patients with advanced dementia may not have been receiving a dementia medication. Whilst these may have resulted in an underestimation of the prevalence of people with dementia, we believe that using both of these approaches to identify study participants helped to mitigate the limitations of using one of these approaches in isolation. The use of electronic health records, such as those used in this study, is limited by the quality and volume of data recorded. The exclusion of those who died during the study period may have contributed to a survivor bias which may have affected the prevalence of PIP; however, we were limited by the data available to us from patients’ electronic records. Finally, the results are based on PwD who visited ambulatory care clinics of a single tertiary hospital in Riyadh; therefore, the findings may not be generalisable to all PwD across Saudi Arabia or different healthcare settings. Future work is needed to corroborate our findings across a more representative sample in Saudi Arabia. Tools such as the STOPP criteria are useful for both alerting healthcare providers to the use of potentially inappropriate medications and monitoring effectiveness for intervention-based studies that aimed to reduce PIP [63]. However, the use of such indicators of prescribing appropriateness should not replace clinical judgment and taking a person-centred approach to patient care.

## Conclusion

Our findings have revealed a high prevalence of PIP that is strongly associated with polypharmacy among PwD in the ambulatory care setting. The perspectives of key stakeholders in medicines management including health care providers, PwD, and their carers in Saudi Arabia need to be explored in future research.

### Electronic supplementary material

Below is the link to the electronic supplementary material.

Supplementary Material 1

Supplementary Material 2

## Data Availability

The data are available from the corresponding author upon reasonable request.

## Abbreviations

ACB: Anticholinergic burden
ATC: Anatomical therapeutic chemical classification
BPSD: Behavioural and psychological symptoms of dementia
EHRs: Electronic health records
NSAID: Non-steroidal anti-inflammatory drugs
PIP: Potentially inappropriate prescribing
PPI: Proton pump inhibitor
PUD: Peptic ulcer disease
PwD: People with dementia
STOPP: Screening Tool of Older Persons Potentially Inappropriate Prescriptions
WHO: World Health Organisation
