# Supplementary material for: Potentially inappropriate prescribing for people with dementia in ambulatory care: a cross-sectional observational study
Source: BMC Geriatr. 2024 Apr 10;24:328. doi: 10.1186/s12877-024-04949-8 (PMC11008018; doi:10.1186/s12877-024-04949-8)
Supplement: Supplementary file 1 — Supplementary Material 1 [file 12877_2024_4949_MOESM1_ESM.docx]

**Additional File 1.** The Strengthening the Reporting of Observational Studies in Epidemiology (STROBE) statement checklist 2008.

|  | **Item No.** | | | **Recommendation** | **Page  No.** |
| --- | --- | --- | --- | --- | --- |
| **Title and abstract** | | 1 | (*a*) Indicate the study’s design with a commonly used term in the title or the abstract | | 1,4 |
|  |  |  | (*b*) Provide in the abstract an informative and balanced summary of what was done and what was found | | 4-5 |
| **Introduction** | | | | | |
| Background/rationale | | 2 | Explain the scientific background and rationale for the investigation being reported | | 6-8 |
| Objectives | | 3 | State specific objectives, including any prespecified hypotheses | | 8 |
| **Methods** | | | | | |
| Study design | | 4 | Present key elements of study design early in the paper | | 9 |
| Setting | | 5 | Describe the setting, locations, and relevant dates, including periods of recruitment, exposure, follow-up, and data collection | | 8-9 |
| Participants | | 6 | (*a*) Give the eligibility criteria, and the sources and methods of selection of participants | | 9-10 |
| Variables | | 7 | Clearly define all outcomes, exposures, predictors, potential confounders, and effect modifiers. Give diagnostic criteria, if applicable | | 10-11 |
| Data sources/ measurement | | 8* | For each variable of interest, give sources of data and details of methods of assessment (measurement). Describe comparability of assessment methods if there is more than one group | | 8-11 |
| Bias | | 9 | Describe any efforts to address potential sources of bias | | N/A |
| Study size | | 10 | Explain how the study size was arrived at | | 12 |
| Quantitative variables | | 11 | Explain how quantitative variables were handled in the analyses. If applicable, describe which groupings were chosen and why | | 12 |
| Statistical methods | | 12 | (*a*) Describe all statistical methods, including those used to control for confounding | | 12 |
|  | |  | (*b*) Describe any methods used to examine subgroups and interactions | | N/A |
|  | |  | (*c*) Explain how missing data were addressed | | N/A |
|  | |  | (*d*) If applicable, describe analytical methods taking account of sampling strategy | | N/A |
|  | |  | (*e*) Describe any sensitivity analyses | | N/A |
| **Results** | |  |  | |  |
| Participants | | 13* | (a) Report numbers of individuals at each stage of study—e.g. numbers potentially eligible, examined for eligibility, confirmed eligible, included in the study, completing follow-up, and analysed | | 12 |
|  | |  | (b) Give reasons for non-participation at each stage | | 12 |
|  | |  | (c) Consider use of a flow diagram | | N/A |
| Descriptive data | | 14* | (a) Give characteristics of study participants (e.g. demographic, clinical, social) and information on exposures and potential confounders | | 12-17 |
|  | |  | (b) Indicate number of participants with missing data for each variable of interest | | N/A |
| Outcome data | | 15* | Report numbers of outcome events or summary measures | | 14-17 |
| Main results | | 16 | (*a*) Give unadjusted estimates and, if applicable, confounder-adjusted estimates and their precision (e.g. 95% confidence interval). Make clear which confounders were adjusted for and why they were included | | 16-17 |
|  | |  | (*b*) Report category boundaries when continuous variables were categorized | | 17 |
|  | |  | (*c*) If relevant, consider translating estimates of relative risk into absolute risk for a meaningful time period | | N/A |
| Other analyses | | 17 | Report other analyses done—e.g. analyses of subgroups and interactions, and sensitivity analyses | | N/A |
| **Discussion** | |  |  | |  |
| Key results | | 18 | Summarise key results with reference to study objectives | | 17 |
| Limitations | | 19 | Discuss limitations of the study, taking into account sources of potential bias or imprecision. Discuss both direction and magnitude of any potential bias | | 21-22 |
| Interpretation | | 20 | Give a cautious overall interpretation of results considering objectives, limitations, multiplicity of analyses, results from similar studies, and other relevant evidence | | 17-22 |
| Generalisability | | 21 | Discuss the generalisability (external validity) of the study results | | 22 |
| **Other information** | |  |  | |  |
| Funding | | 22 | Give the source of funding and the role of the funders for the present study and, if applicable, for the original study on which the present article is based | | 2 |
