# Supplementary material for: Potentially inappropriate prescribing for people with dementia in ambulatory care: a cross-sectional observational study
Source: BMC Geriatr. 2024 Apr 10;24:328. doi: 10.1186/s12877-024-04949-8 (PMC11008018; doi:10.1186/s12877-024-04949-8)
Supplement: Supplementary file 2 — Supplementary Material 2 [file 12877_2024_4949_MOESM2_ESM.docx]

**Additional file 2**. Prevalence of potentially inappropriate prescribing among 287 people with dementia by individual STOPP Criterion.

| STOPP criterion description | Number of patients | % of patients |
| --- | --- | --- |
| A: Indication of medication | | |
| Any drug prescribed without an evidence-based clinical indication.      PPIs       Diuretics       Factor Xa inhibitors       SSRIs       Pentoxifylline       Domperidone | 78  65  5  3  3  1  1 | 27.2  22.6  1.7  1.0  1.0  0.3  0.3 |
| Any drug prescribed beyond the recommended duration, where treatment duration is well defined.  PPIs       Melatonin       NSAIDs       Zolpidem | 90  75  10  4  1 | 31.4  26.0  3.5  1.4  0.3 |
| Any duplicate drug class prescription *(optimisation of monotherapy within a single drug class should be observed prior to considering a new agent)*    CCBs     AchEIs     BBs     Antipsychotics     SSRIs     Alpha blockers     Thiazide diuretics     Sulphonylurea | 23  6  4  3  3  2  2  2  1 | 8.0  2.1  1.4  1.0  1.0  0.7  0.7  0.7  0.3 |
| B: Cardiovascular drugs | | |
| Digoxin for heart failure with normal systolic ventricular function *(no clear evidence of benefit)* | 0 | 0 |
| Verapamil or diltiazem with NYHA class III or IV heart failure (*may worsen heart failure)* | 1 | 0.4 |
| BB in combination with verapamil or diltiazem *(risk of heart block)* | 0 | 0 |
| BB with bradycardia (< 50/min), type II heart block or complete heart block *(risk of complete heart block, asystole)* | 0 | 0 |
| Amiodarone as first-line antiarrhythmic therapy in supraventricular tachyarrhythmias *(higher risk of side-effects than beta-blockers, digoxin, verapamil, or diltiazem)* | 0 | 0 |
| Loop diuretic as first-line treatment for hypertension *(safer, more effective alternatives available)* | 1 | 0.4 |
| Loop diuretic for dependent ankle oedema without clinical, biochemical evidence or radiological evidence of heart failure, liver failure, nephrotic syndrome, or renal failure *(leg elevation and /or compression hosiery usually more appropriate)* | 1 | 0.4 |
| Thiazide diuretic with current significant hypokalaemia (i.e., serum K+ < 3.0 mmol/l), hyponatraemia (i.e. serum Na+ < 130 mmol/l) hypercalcaemia (i.e. corrected serum calcium > 2.65 mmol/l) or with a history of gout (*hypokalaemia, hyponatraemia, hypercalcaemia, and gout can be precipitated by thiazide diuretic)* | 0 | 0 |
| Loop diuretic for treatment of hypertension with concurrent urinary incontinence *(may exacerbate incontinence)* | 0 | 0 |
| Centrally acting antihypertensives, unless clear intolerance of, or lack of efficacy with, other classes of antihypertensives (*less well tolerated by older people than younger people*) | 0 | 0 |
| ACE inhibitor or ARB in patients with hyperkalaemia | 0 | 0 |
| Aldosterone antagonists with concurrent potassium conserving drugs (e.g. ACE inhibitor, ARB, amiloride, triamterene) without monitoring of serum potassium *(risk of dangerous hyperkalaemia i.e., > 6.0 mmol/l)* | 0 | 0 |
| Phosphodiesterase type-5 inhibitors in severe heart failure characterised by hypotension i.e., systolic BP < 90 mmHg, or concurrent nitrate therapy for angina *(risk of cardiovascular collapse)* | 0 | 0 |
| C: Antiplatelet/Anticoagulant drugs | | |
| Long-term aspirin at doses greater than 160mg per day *(increased risk of bleeding, no evidence for increased efficacy)* | 0 | 0 |
| Aspirin with a history of PUD without concomitant PPI *(risk of recurrent peptic ulcer)* | 1 | 0.4 |
| Aspirin, clopidogrel, dipyridamole, vitamin K antagonists, direct thrombin inhibitors or factor Xa inhibitors with concurrent significant bleeding risk, i.e., uncontrolled severe hypertension, bleeding diathesis, recent non-trivial spontaneous bleeding) *(high risk of bleeding)* | 0 | 0 |
| Aspirin plus clopidogrel as secondary stroke prevention unless the patient has a coronary stent(s) inserted in the previous 12 months or concurrent acute coronary syndrome or has a high grade symptomatic carotid arterial stenosis (*no evidence of added benefit over clopidogrel monotherapy*) | 15 | 5.2 |
| Aspirin in combination with vitamin K antagonist, direct thrombin inhibitor or factor Xa inhibitors in patients with chronic atrial fibrillation *(no added benefit from aspirin)* | 3 | 1.0 |
| Antiplatelet agents with vitamin K antagonist, direct thrombin inhibitor or factor Xa inhibitors in patients with stable coronary, cerebrovascular or peripheral arterial disease *(no added benefit from dual therapy)* | 2 | 0.7 |
| Vitamin K antagonist, direct thrombin inhibitor or factor Xa inhibitors for first deep venous thrombosis without continuing provoking risk factors (e.g. thrombophilia) for > 6 months, (no proven added benefit) | 0 | 0 |
| Vitamin K antagonist, direct thrombin inhibitor or factor Xa inhibitors for first pulmonary embolus without continuing provoking risk factors (e.g. thrombophilia) for > 12 months (no proven added benefit) | 0 | 0 |
| NSAIDs and vitamin K antagonist, direct thrombin inhibitor or factor Xa inhibitors in combination *(risk of major gastrointestinal bleeding)* | 1 | 0.4 |
| NSAID with concurrent antiplatelet agent(s) without PPI prophylaxis *(increased risk of PUD)* | 2 | 0.7 |
| D: Central nervous system and psychotropic drugs | | |
| TCAs with dementia, narrow angle glaucoma, cardiac conduction abnormalities, prostatism, or prior history of urinary retention *(risk of worsening these conditions)* | 1 | 0.4 |
| Initiation of TCAs as first-line antidepressant treatment *(higher risk of adverse drug reactions with TCAs than with SSRIs or SNRIs)* | 0 | 0 |
| Neuroleptics with moderate-marked antimuscarinic/anticholinergic effects (chlorpromazine, clozapine, flupenthixol, fluphenzine, pipothiazine, promazine, zuclopenthixol) with a history of prostatism or previous urinary retention *(high risk of urinary retention).* | 2 | 0.7 |
| SSRIs with current or recent significant hyponatraemia i.e., serum Na+ < 130 mmol/l *(risk of exacerbating or precipitating hyponatraemia)* | 0 | 0 |
| Benzodiazepines for ≥4 weeks *(no indication for longer treatment; risk of prolonged sedation, confusion, impaired balance, falls, road traffic accidents; all benzodiazepines should be withdrawn gradually if taken for more than 4 weeks as there is a risk of causing a benzodiazepine withdrawal syndrome if stopped abruptly)* | 0 | 0 |
| Antipsychotics (i.e., other than quetiapine or clozapine) in those with parkinsonism or Lewy Body Disease *(risk of severe extrapyramidal symptoms)* | 3 | 1.0 |
| Anticholinergics/ antimuscarinics to treat extrapyramidal side-effects of neuroleptic medications *(risk of anticholinergic toxicity)* | 0 | 0 |
| Anticholinergics/ antimuscarinics in patients with delirium or dementia *(risk of exacerbation of cognitive impairment)* | 28 | 9.8 |
| Neuroleptic antipsychotic in patients with behavioural and psychological symptoms of dementia (BPSD) unless symptoms are severe and other non-pharmacological treatments have failed *(increased risk of stroke).* | 7 | 2.4 |
| Neuroleptics as hypnotics, unless sleep disorder is due to psychosis or dementia (*risk of confusion, hypotension, extra-pyramidal side effects, falls).* | 0 | 0 |
| AchEIs with a known history of persistent bradycardia (< 60 beats/min.), heart block or recurrent unexplained syncope or concurrent treatment with drugs that reduce heart rate such as BBs, digoxin, diltiazem, verapamil *(risk of cardiac conduction failure, syncope, and injury)* | 60 | 21.0 |
| Phenothiazines as first-line treatment, since safer and more efficacious alternatives exist *(phenothiazines are sedative, have significant anti-muscarinic toxicity in older people, with the exception of prochlorperazine for nausea/vomiting/vertigo, chlorpromazine for relief of persistent hiccoughs and levomepromazine as an anti-emetic in palliative care)* | 0 | 0 |
| Levodopa or dopamine agonists for benign essential tremor *(no evidence of efficacy)* | 0 | 0 |
| First generation antihistamines *(safer, less toxic antihistamines now widely available)* | 0 | 0 |
| E: Renal System. The following drugs are potentially inappropriate in older people with acute or chronic kidney disease with renal function below particular levels of eGFR | | |
| Digoxin at a long-term dose greater than 125µg/day if eGFR < 30 ml/min/1.73m2 *(risk of digoxin toxicity if plasma levels not measured)* | 0 | 0 |
| Direct thrombin inhibitors if eGFR < 30 ml/min/1.73m2 *(risk of bleeding)* | 0 | 0 |
| Factor Xa inhibitors if eGFR < 15 ml/min/1.73m2 *(risk of bleeding)* | 0 | 0 |
| NSAID’s if eGFR < 50 ml/min/1.73m2 *(risk of deterioration in renal function)* | 0 | 0 |
| Colchicine if eGFR < 10 ml/min/1.73m2 *(risk of colchicine toxicity)* | 0 | 0 |
| Metformin if eGFR < 30 ml/min/1.73m2 *(risk of lactic acidosis)* | 2 | 0.7 |
| F: Gastro-intestinal system | | |
| Prochlorperazine or metoclopramide with Parkinsonism *(risk of exacerbating Parkinsonian symptoms)* | 0 | 0 |
| PPI for uncomplicated PUD or erosive peptic oesophagitis at full therapeutic dosage for >8 weeks *(dose reduction or earlier discontinuation indicated)* | 75 | 26.0 |
| Drugs likely to cause constipation (e.g. antimuscarinic/anticholinergic drugs, oral iron, opioids, verapamil, aluminium antacids) in patients with chronic constipation where non constipating alternatives are available *(risk of exacerbation of constipation)* | 3 | 1.0 |
| Oral elemental iron doses greater than 200mg daily *(no evidence of enhanced iron absorption above these doses)* | 0 | 0 |
| G: Respiratory system | | |
| Theophylline as monotherapy for COPD *(safer, more effective alternative; risk of adverse effects due to narrow therapeutic index)* | 0 | 0 |
| Systemic corticosteroids instead of inhaled corticosteroids for maintenance therapy in moderate-severe COPD *(unnecessary exposure to long-term side-effects of systemic corticosteroids and effective inhaled therapies are available)* | 0 | 0 |
| Antimuscarinic bronchodilators with a history of narrow angle glaucoma *(may exacerbate glaucoma)* or bladder outflow obstruction *(may cause urinary retention)* | 3 | 1.0 |
| Non-selective BB (whether oral or topical for glaucoma) with a history of asthma requiring treatment *(risk of increased bronchospasm)* | 0 | 0 |
| Benzodiazepines with acute or chronic respiratory failure i.e. pO2 < 8.0 kPa ± pCO2 > 6.5 kPa (risk of exacerbation of respiratory failure) | 1 | 0.4 |
| H: Musculoskeletal system | | |
| NSAID other than COX-2 selective agents with history of PUD or gastrointestinal bleeding, unless with concurrent PPI or H2 antagonist (risk of peptic ulcer relapse) | 2 | 0.7 |
| NSAID with severe hypertension *(risk of exacerbation of hypertension)* or severe heart failure *(risk of exacerbation of heart failure)* | 1 | 0.4 |
| Long-term use of NSAID (>3 months) for symptom relief of osteoarthritis pain where paracetamol has not been tried *(simple analgesics preferable and usually as effective for pain relief)* | 1 | 0.4 |
| Long-term corticosteroids (>3 months) as monotherapy for rheumatoid arthritis *(risk of systemic corticosteroid side-effects)* | 0 | 0 |
| Corticosteroids (other than periodic intra-articular injections for mono-articular pain) for osteoarthritis *(risk of systemic corticosteroid side-effects)* | 0 | 0 |
| Long-term NSAID or colchicine (>3 months) for chronic treatment of gout where there is no contraindication to a xanthine-oxidase inhibitor (e.g. allopurinol, febuxostat) *(xanthine-oxidase inhibitors are first choice prophylactic drugs in gout)* | 0 | 0 |
| COX-2 selective NSAID with concurrent cardiovascular disease *(increased risk of myocardial infarction and stroke)* | 2 | 0.7 |
| NSAID with concurrent corticosteroids without PPI prophylaxis *(increased risk of peptic ulcer disease)* | 0 | 0 |
| Oral bisphosphonates in patients with a current or recent history of upper gastrointestinal disease i.e., dysphagia, oesophagitis, gastritis, duodenitis, or PUD, or upper gastrointestinal bleeding *(risk of relapse/exacerbation of oesophagitis, oesophageal ulcer, oesophageal stricture)* | 4 | 1.4 |
| I: Urogenital system | | |
| Antimuscarinic drugs with dementia, or chronic cognitive impairment *(risk of increased confusion)* or narrow-angle glaucoma *(risk of acute exacerbation of glaucoma)* or chronic prostatism *(risk of urinary retention)* | 25 | 8.7 |
| Selective alpha-1 selective alpha blockers in those with symptomatic orthostatic hypotension or micturition syncope *(risk of precipitating recurrent syncope).* | 0 | 0 |
| J: Endocrine system | | |
| Sulfonylureas with a long duration of action with type 2 diabetes mellitus *(risk of prolonged hypoglycaemia)* | 12 | 4.2 |
| Thiazolidinediones in patients with heart failure *(risk of exacerbation of heart failure)* | 0 | 0 |
| Oestrogens with a history of breast cancer or venous thromboembolism (*increased risk of recurrence).* | 0 | 0 |
| Oral oestrogens without progestogen in patients with intact uterus *(risk of endometrial cancer)* | 0 | 0 |
| Androgens (male sex hormones) in the absence of primary or secondary hypogonadism *(risk of androgen toxicity; no proven benefit outside of the hypogonadism indication).* | 0 | 0 |
| K: Drugs that predictably increase the risk of falls in older people | | |
| Benzodiazepines *(sedative, may cause reduced sensorium, impair balance)* | 3 | 1.0 |
| Neuroleptic drugs *(may cause gait dyspraxia, Parkinsonism)* | 1 | 0.4 |
| Hypnotic Z-drugs *(may cause protracted daytime sedation, ataxia).* | 1 | 0.4 |
| L: Analgesic drugs | | |
| Use of oral or transdermal strong opioids as first-line therapy for mild pain *(WHO analgesic ladder not observed)* | 0 | 0 |
| Use of regular (as distinct from PRN) opioids without concomitant laxative *(risk of severe constipation)* | 1 | 0.4 |
| Long-acting opioids without short-acting opioids for breakthrough pain *(risk of persistence of severe pain)* | 0 | 0 |
| M: Antimuscarinic/Anticholinergic drug burden | | |
| Concomitant use of two or more drugs with antimuscarinic/anticholinergic properties *(risk of increased antimuscarinic/anticholinergic toxicity)* | 2 | 0.7 |

AchEIs, acetylcholine esterase inhibitors;  ACE, angiotensin converting enzyme; ARBs, angiotensin receptors blockers; BBs, betablockers; BP, blood pressure; CCBs, calcium channel blockers; COPD, chronic obstructive pulmonary disease; COX-2, cyclooxygenase-2; eGFR, estimated glomerular filtration rate;  NSAID, non-steroidal anti-inflammatory drug; NYHA, New York heart association; PPI, proton pump inhibitor; PRN, when required; PUD, peptic ulcer disease; SSRI, selective serotonin reuptake inhibitor; STOPP, Screening Tool of Older Persons Potentially Inappropriate Prescriptions; TCA, tricyclic antidepressant; WHO, World Health Organization
